# Surgical Management of Complex Multiligament Knee Injury: Case Report

**DOI:** 10.1155/2024/2594659

**Published:** 2024-10-19

**Authors:** Ramon Alonso Prieto Baeza, Fernando González González, Fernando Hernández Aragon, David Alfonso Servín Pérez, Nadia Karina Portillo Ortiz, Andrés Manuel García Carrera, Arturo Aguirre Madrid, Edmundo Berumen Nafarrate

**Affiliations:** ^1^Orthopedic Surgeon, Star Médica Chihuahua Hospital, Perif. de la Juventud 6103, Fracc. El Saucito 31110, Chihuahua, Chihuahua, Mexico; ^2^Orthopedic Surgeon, Christus Muguerza del Parque Hospital, de la Llave St. No. 1419, Office 9, Col. Centro 31000, Chihuahua, Chihuahua, Mexico; ^3^Faculty of Medicine and Biomedical Sciences, University Autonomous of Chihuahua, Chihuahua, Mexico; ^4^Faculty of Medicine and Biomedical Sciences, University of Durango, Chihuahua, Mexico; ^5^Department of Orthopedic Surgery, Star Medica Chihuahua Hospital, Perif. de la Juventud 6103, Fracc. El Saucito, Chihuahua, Chihuahua 31110, Mexico; ^6^Academic Division, Star Medica Chihuahua Hospital, Perif. de la Juventud 6103, Fracc. El Saucito, Chihuahua, Chihuahua 31110, Mexico

**Keywords:** anatomical reconstruction, double-bundle technique, knee stability, multiligament knee injury

## Abstract

Multiligament knee injuries (MLKIs) frequently require immediate intervention to prevent severe complications, including vascular injury. We present the case of a 51-year-old male who sustained a traumatic right knee dislocation following a motor vehicle accident. The patient exhibited significant tibiofemoral dissociation with Grade 3 instability, classified as Schenck KD IV. Immediate reduction and external fixation were performed, followed by definitive surgical management, which included fibular sling, MPFL and MCL repair, and double-bundle and double-tunnel ACL and PCL reconstruction with looped proximal tibial fixation. The patient showed an excellent early postoperative outcome, with minimal edema, manageable moderate pain, and a full range of motion by the 30-day follow-up. This case underscores the effectiveness of combining fibular sling, MPFL, and MCL, with anatomical double-bundle ACL and PCL reconstruction in the treatment of complex MLKIs. The level of evidence is IV.

## 1. Introduction

Multiligament knee injuries (MLKIs) represent a severe and complex pathology defined as injuries to at least two of the four major knee ligaments: the anterior cruciate ligament, posterior cruciate ligament, medial collateral ligament (including the posteromedial corner), and lateral collateral ligament (including the posterolateral (PL) corner). MLKIs can result from high-energy trauma, often associated with other injuries, or from low-energy trauma, such as knee dislocations in obese patients following a fall from height [1–3].

The complexity of MLKIs emphasizes the need for a thorough clinical assessment, including a thorough clinical history, comprehensive physical examination, and advanced radiographic evaluation. Acute MLKIs often involve high-energy trauma, requiring assessment using advanced trauma life support principles (ATLS), while subacute and chronic MLKIs will depend on specific tests for the ACL, PCL, MCL, and LCL, like the anterior drawer, Lachman, pivot shift, and dial tests. Additionally, clinical analysis using digital arthrometry such as the KT1000 and KT2000 arthrometers (MED metric Corp., San Diego, CA, United States) provides an accurate and objective measurement of knee laxity. A smartphone application called “Pivot-Shift Meter (PSM)” has been developed, leveraging the gyroscopes and accelerometers integrated into mobile phones to allow for an accurate and numerical assessment of knee rotational instability. This technological advancement aids clinicians in obtaining precise data on knee stability, enhancing the overall evaluation and treatment planning for patients with MLKIs [4, 5].

Schenck's classification, based on clinical and radiologic assessments, is the most commonly used classification for these injuries. It was initially described for knee dislocations and later modified by Wascher to include fracture dislocations. (Table 1) [6].

Approximately 18% of MLKIs are complicated by vascular injury, emphasizing the importance of early and thorough assessment to mitigate potential limb-threatening consequences. The management of these injuries poses a significant challenge due association of potential vascular compromise and concurrent soft tissue damage. Inadequate diagnosis and treatment can lead to persistent pain, instability, and underwhelming functional recovery [2, 7–10].

Surgical intervention, particularly within the acute phase, has emerged as the preferred approach, demonstrating superior patient-reported outcomes, functional recovery, and return to preinjury activities compared to nonoperative methods. Early surgical intervention, typically within 3 weeks postinjury, is advocated to capitalize on favorable outcomes and prevent residual instability. However, the optimal timing of surgery remains controversial, with some studies suggesting delayed intervention may reduce complications in certain patient populations [11, 12].

While surgical management is often the preferred treatment for multiligamentous knee injuries, surgeons must be aware of risk factors, such as age, tourniquet time dilation, previous knee surgeries, smoking, and higher BMI, that may determine postoperative outcomes and complications including wound infection (up to 12.5%), deep venous thrombosis (DVT, up to 17%), and arthrofibrosis (8%–9%) [13–16].

This report details the case of a patient presenting a Schenck KD IV MLKI, associated with soft tissue structures of the right knee. The surgical management, utilizing fibular sling, MPFL, MCL reconstruction, and anatomical U2 double-bundle reconstruction for the ACL and PCL, is described, highlighting the rationale and considerations for treatment selection.

## 2. Clinical Case

**Figure 1:**
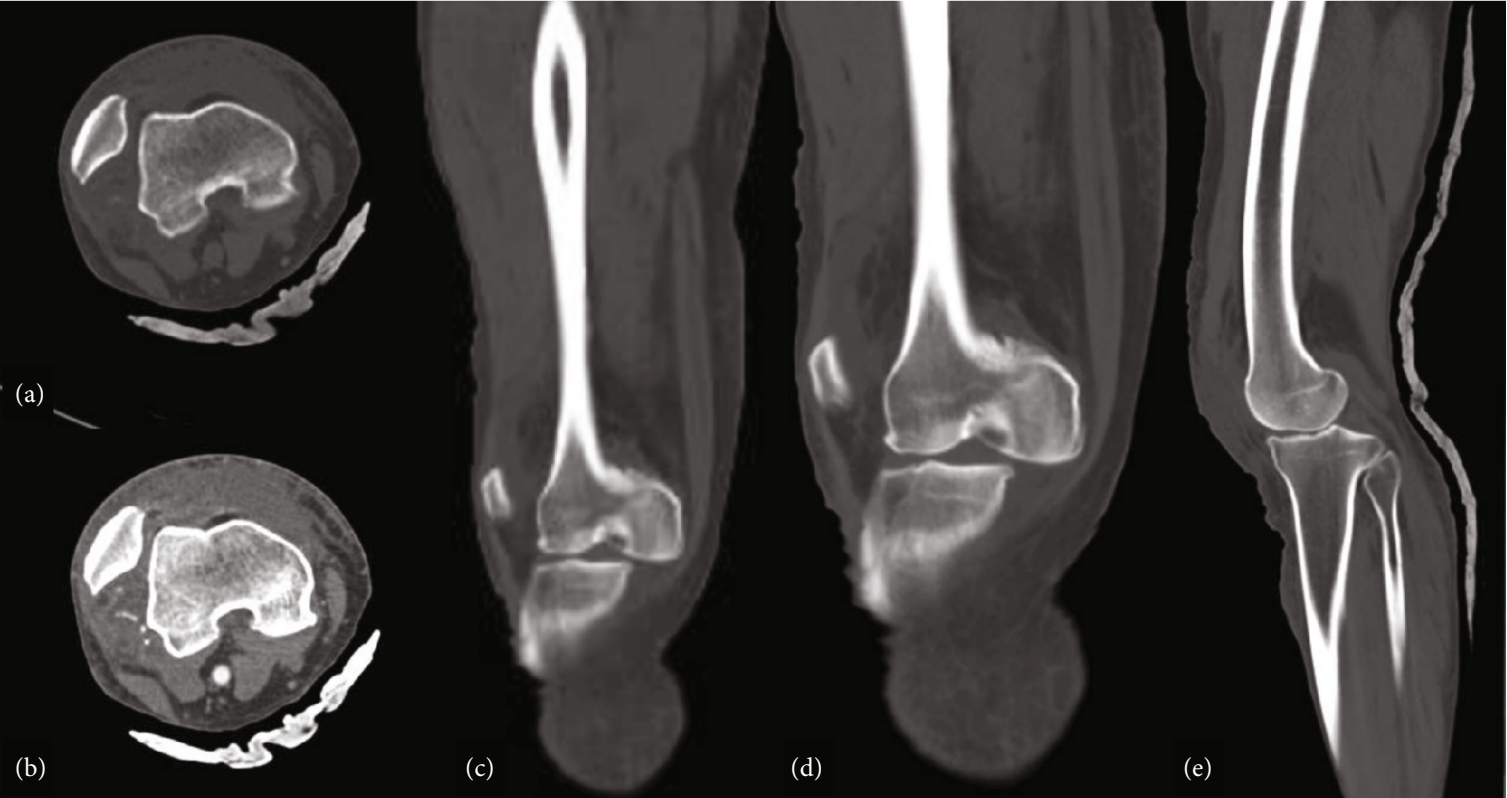
(a, b) Axial, (c, d) AP, and (e) lateral CT-scan: dislocation of the femorotibial joint is observed, with medial displacement of the femoral portion. Additionally, an avulsion fracture is identified on the lateral tibial surface, specifically in its medial portion. Furthermore, lateral displacement of the patella is noted. The quadriceps and patellar tendons exhibit normal density.

**Figure 2:**
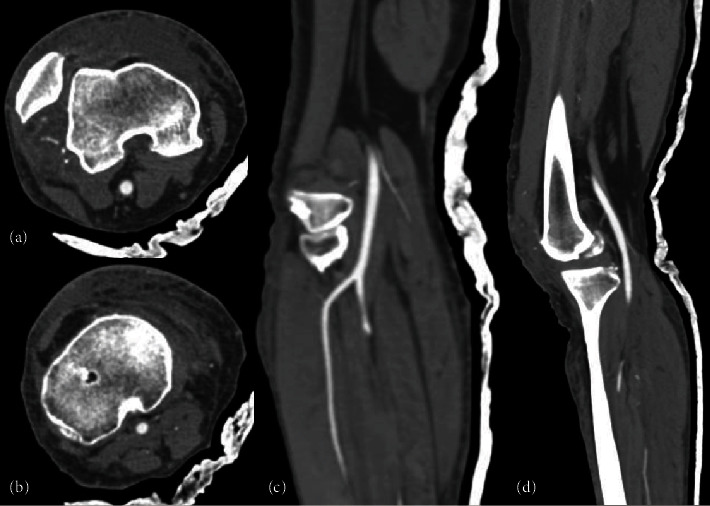
Angiotomography results. (a, b) Axial and (c, d) lateral view: the common femoral vein, superficial femoral vein, and deep femoral vein are intact.

A 51-year-old male with no significant medical history presented with a complete dislocation of the right knee following a motor vehicle accident. Paramedics provided prehospital care and were transported via an ambulance to the hospital. Upon admission, the emergency department evaluated the patient, and vital signs were as follows: blood pressure of 130/64, temperature of 36°C, respiratory rate of 18, heart rate of 95, and Glasgow Coma Scale score of 14. During the physical inspection, a severe deformity in the valgus was noted in the right knee, along with intense pain and limited motion. An urgent CT angiogram scan of the right lower limb was performed, revealing a traumatic PL knee dislocation without vascular or nervous injury, along with sagittal and coronal instability (Figures 1 and 2).

**Figure 3:**
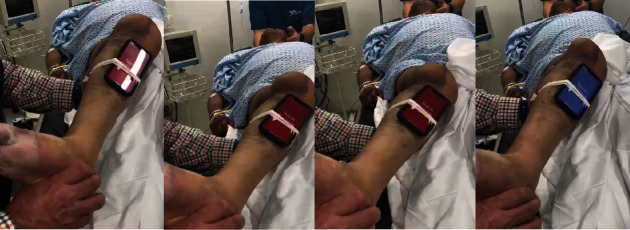
Physical examination performed on anesthetized patient prior to external fixation involving a combination of rotational and valgus forces, making it sensitive to both rotational and anterior-posterior stability, with a primary plane of motion in the transverse plane. Pivot shift maneuver observed from left to right is presented, with clear tibiofemoral dissociation. The PSM app was utilized to calculate the degree of instability using quantitative data, yielding a result of Grade 3 instability.

The patient was programmed for urgent reduction under sedation in the emergency department, and a detailed physical examination of the knee was performed prior to reduction, noting clear signs of tibiofemoral dissociation. Additionally, the PSM app was employed to rapidly obtain quantitative data on knee instability. This tool utilizes mobile inertial sensors to measure and classify the pivot shift phenomenon, offering a precise and objective assessment of rotational instability. The app's validated algorithms analyze the dynamic movement of the knee, providing real-time data on the degree of instability [17]. In this case, the PSM app identified a marked Grade 3 instability, confirming severe rotational laxity, which was crucial in guiding the subsequent surgical management (Figure 3).

**Figure 4:**
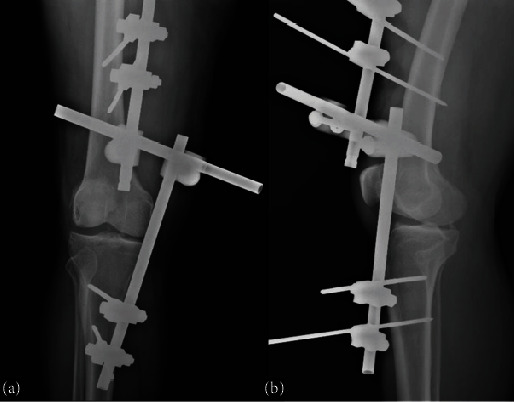
X-ray: (a) AP and (b) lateral of the right knee post external fixation.

The patient was transferred to the operating room to undergo external fixation to address the acute instability and provide temporary stabilization until the definitive surgical procedure could be planned and performed (Figure 4).

**Figure 5:**
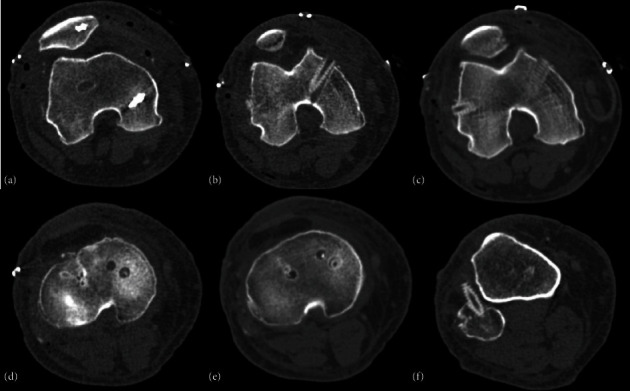
CT scan following multiligamentous reconstruction demonstrates adequate reduction of dislocation. (a–c) tunnel trajectory is identified as coursing caudocephalically toward the lateral femoral condyle, with an oblique disposition. Additionally, there is discreetly oblique cephalocaudal tunneling of the medial femoral condyle, reaching the inner portion of the said condyle. (d, e) Oblique tunneling of the internal tibial plateau is noted, with bone fragments within; slightly medial to this trajectory, a second tunnel is partially occupied by a screw. (f) At the level of the proximal epiphysis of the fibula, complete anteroposterior tunneling is identified, partially occupied by a fixation screw.

**Figure 6:**
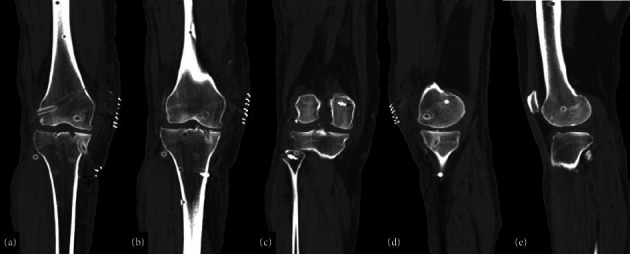
The proximal (a–d) tibiofibular and femorotibial and (e) patellofemoral joints show adequate congruence without alterations in the width of their joint spaces.

Following a definitive diagnosis, the surgical procedure involved ACL, PCL, MPFL, MCL, and PLC reconstruction (Figures 5 and 6).

## 3. Graft Harvest and Preparation

In this case, we selected grafts with a length of 25–30 cm and a width of 8–10 mm per bundle, which is the preferred size for ensuring sufficient tissue for effective ligament reconstruction. Both grafts were obtained from the “Tissue Bank of Guadalajara,” contributing to the comprehensive surgical strategy aimed at restoring the stability and functionality of the patient's knee joint. The allografts were meticulously prepared with Krackow sutures at both ends to ensure secure fixation during the surgical procedure. This preparation method enhances postoperative stability and function by ensuring that the grafts are robustly anchored and properly sized for the reconstruction.

### 3.1. Double Bundle U-DOS Technique for ACL Reconstruction

**Figure 7:**
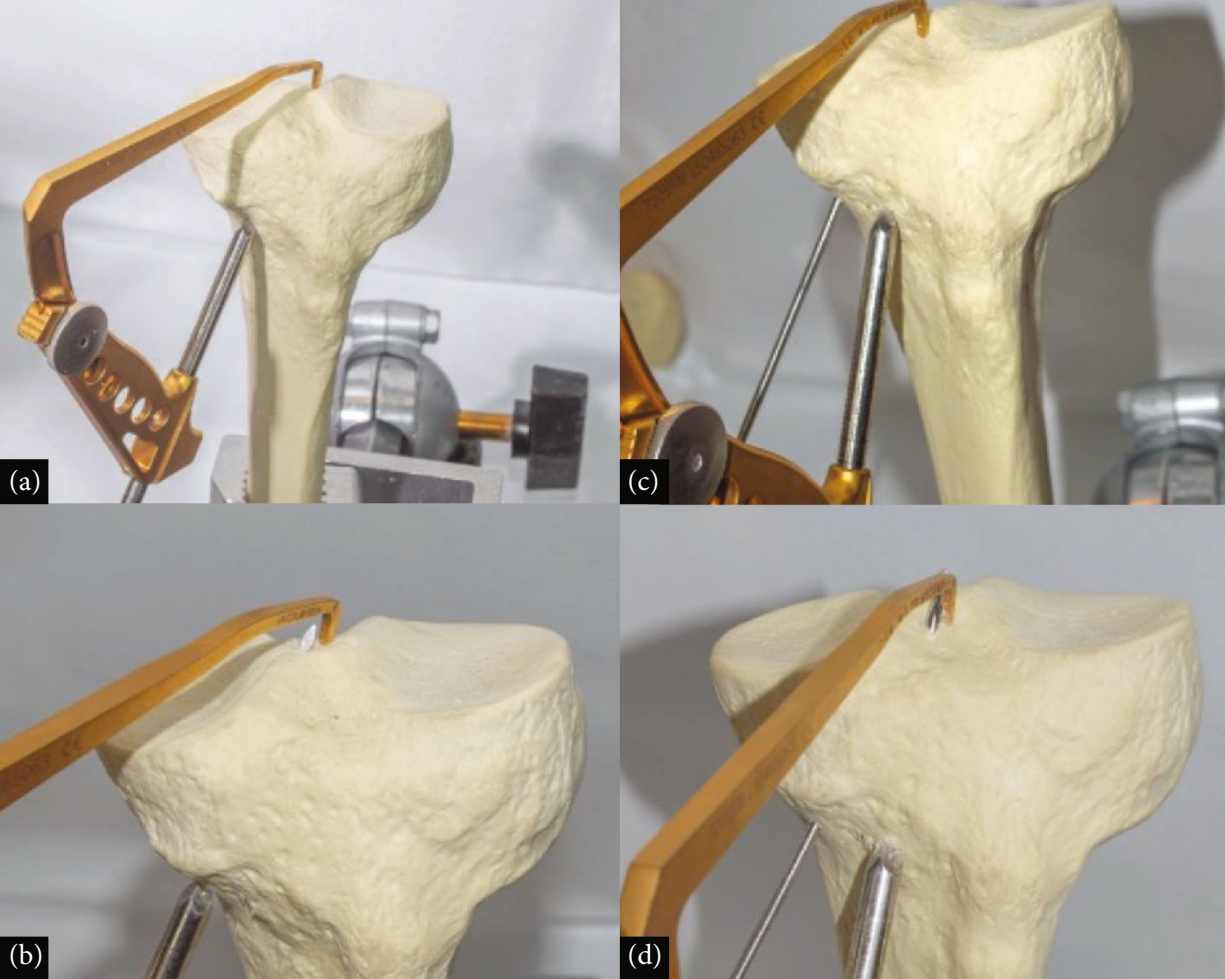
Placement of guide and brocade for the transtibial tunnel for anterior cruciate with double bundle. Bundles: (a, b) AM and (c, d) PL. The tibial tunnels are directed from the anteromedial side of the proximal metaphysis. Both lay at the same level, located 1 cm behind the anterior tibial tuberosity and 1 cm from each other. The intra-articular exit of the tunnels is arthroscopically monitored and located 8 mm anterior to the PCL, at the original ACL footprint.

The ACL reconstruction was performed using a standard arthroscopic approach. An arthroscopic ruler was inserted through the anterolateral portal to determine the optimal positions for both femoral and tibial tunnels. For precise tunnel placement, we targeted the anteromedial (AM) bundle tunnel at the lower part of the native ACL footprint and the PL bundle tunnel just anterior to the posterior wall. These locations were chosen to replicate the natural ACL anatomy and improve the biomechanical function of the knee joint (Figure 7).

**Figure 8:**
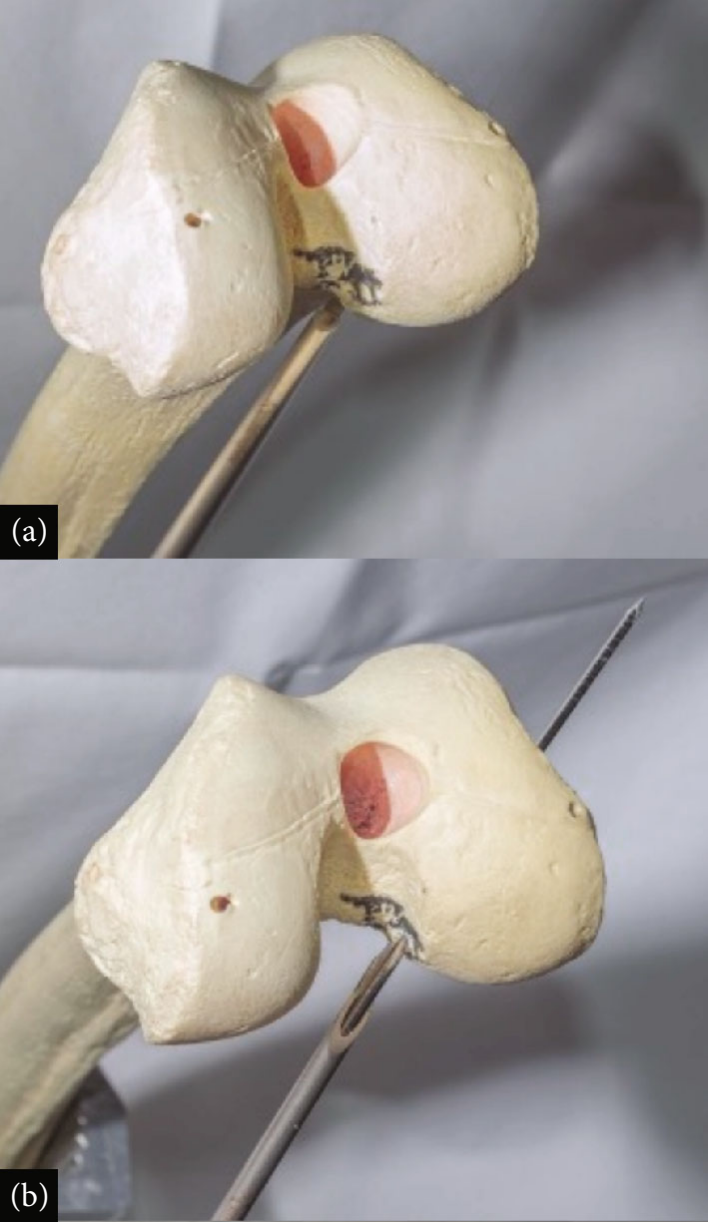
Drilling of the femoral tunnels. The resident's ridge is identified as (a) the anterior boundary, with the over-the-top edge serving as (b) the posterior boundary. The femoral tunnels are then strategically positioned within the upper and lower spaces defined by these anatomical landmarks.

Using a transtibial approach, an intra-articular femoral guide was positioned over the ACL footprint. The first femoral tunnel for the AM bundle was drilled in the central region of its footprint, ensuring anatomic placement while avoiding the posterior wall. The second tunnel for the PL bundle was drilled, ensuring accurate positioning relative to the posterior cortical wall, but anterior enough to prevent blowout, enhancing graft stability (Figure 8).

**Figure 9:**
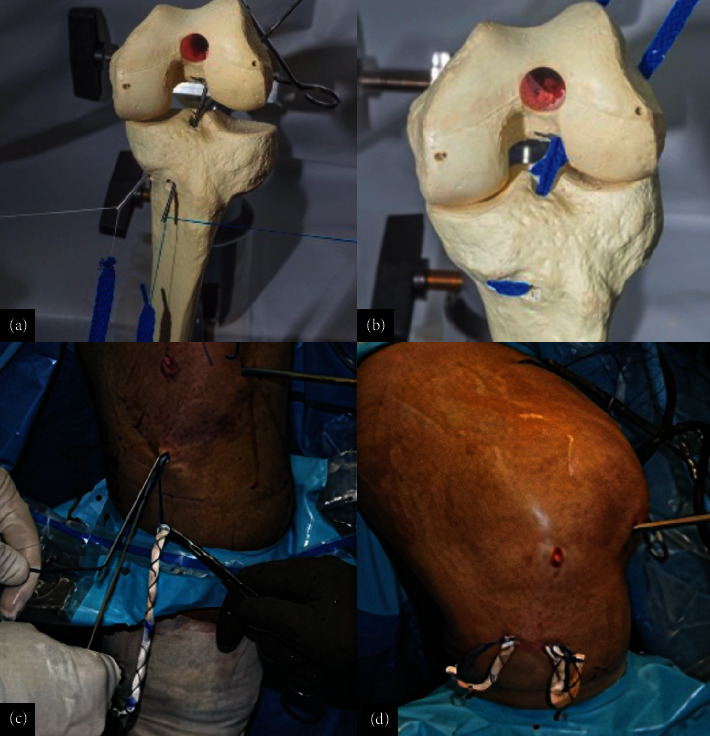
Representation in an anatomical model of (a, b) graft passage and (c, d) intraoperative image. The graft is pulled in from distal to proximal, and looped on the tibial medial proximal cortex. No fixation is performed on the tibia, and the graft should pass easily for similar lengths on both bundles. The posterolateral bundle is first fixed, and the graft is taken into maximum tension for the AM bundle fixation.

The graft was then anchored proximally and pulled through the tibial and femoral tunnels for optimal placement. To achieve optimal graft tension, intraoperative tensioning was meticulously performed by preconditioning the grafts with a controlled force before fixation, ensuring that the AM bundle was tensioned in full extension and the PL bundle was tensioned in slight flexion of approximately 30° (Figure 9). This method aligns with biomechanical principles that replicate native ACL tension patterns throughout the range of motion, improving stability and reducing the risk of overconstraint or laxity.

**Figure 10:**
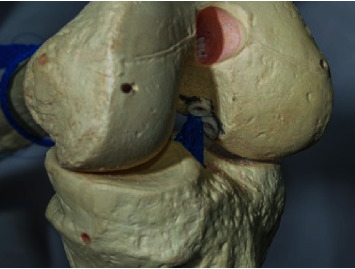
Schematic view of the double-bundle and double-tunnel ACL reconstruction with looped proximal tibial fixation procedure on a model. The U-shaped anchorage is on the tibia and the dual fixation is on the femur. This is an anatomical double-bundle double-tunnel reconstruction with no tibial hardware.

Fixation was achieved using bioabsorbable interference screws, with graft tension carefully adjusted before final fixation to achieve optimal knee stability. The tibial fixation was achieved through a natural U-shaped trajectory, obviating the need for additional hardware, which not only simplified the procedure but also reduced potential hardware-related complications (Figure 10).

### 3.2. Double Bundle U-DOS Reverse Technique for PCL Reconstruction

**Figure 11:**
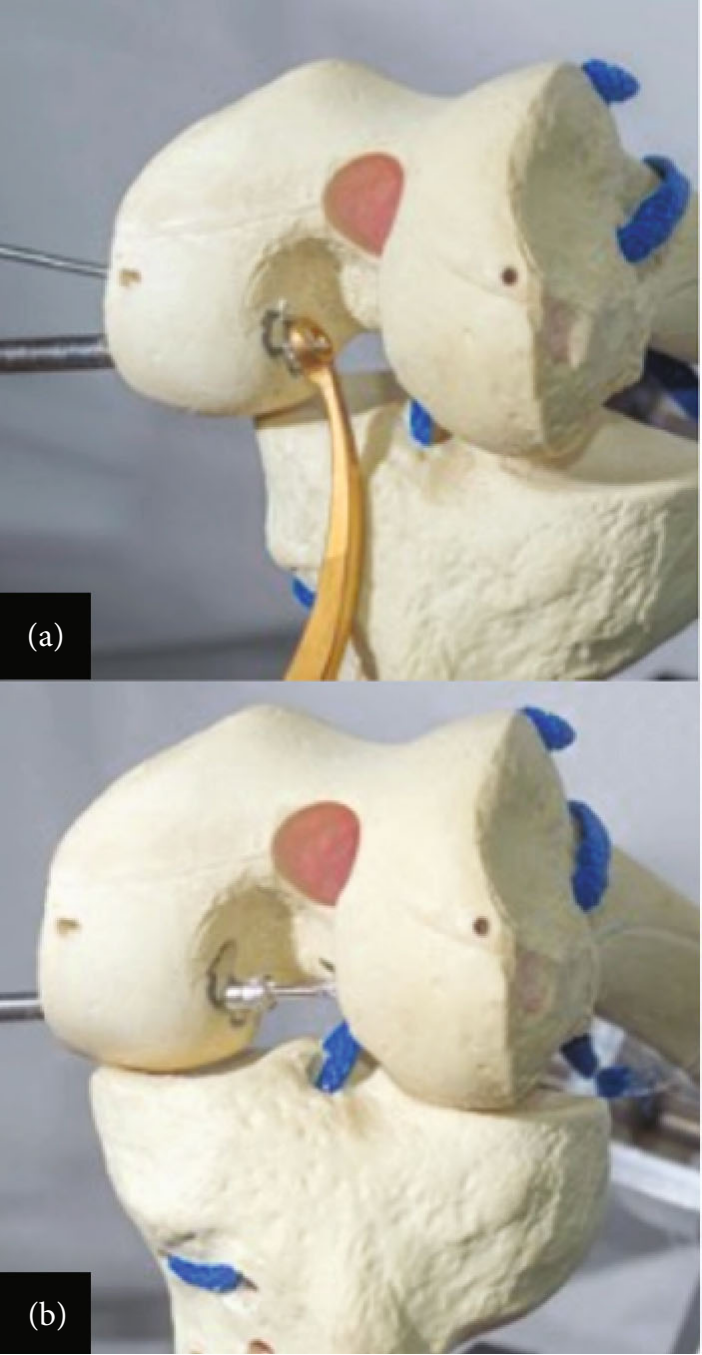
Passage of guides in the femoral condyle with a Clancy system in an (a) out–in manner, with location and exit in the footprint of the PCL at its (b) femoral insertion.

The double bundle U-DOS reverse technique for PCL is an advanced arthroscopic procedure designed to enhance posterior stability and functional outcomes by closely replicating the natural anatomy of the PCL. In this case, the patient was positioned supine with the knee flexed at 70°–80°, and working portals were created with particular attention to the posteromedial portal for visualization and preparation of the tibial insertion of the PCL. Inferomedial and inferolateral parapatellar portals, along with a superomedial exit portal, were established, with additional posteromedial portals aiding in visualization. A Clancy system was employed to guide the drilling of femoral tunnels in an outside–in manner at two points corresponding to the PCL insertion on the femoral condyle (Figure 11).

**Figure 12:**
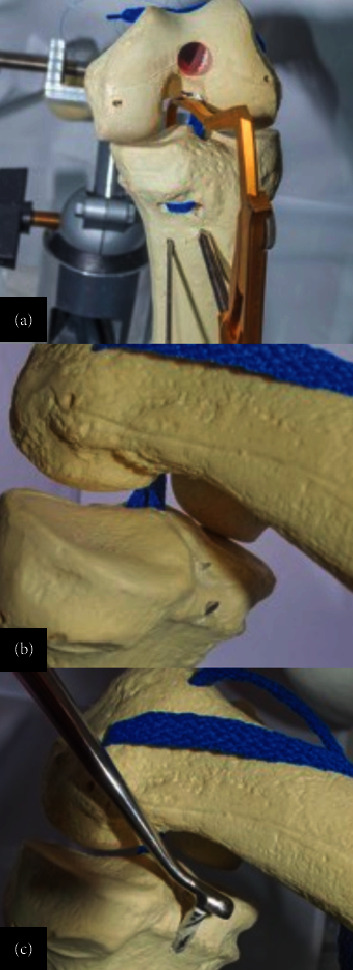
(a, b) Broaching and passage of guides through tunnels in the posterior area of the tibia. (c) 0.5 inches inferior to the articular surface with the use of a posterior guide.

Similarly, tunnels were drilled in the posterior tibia, approximately 1–1.5 cm below the articular surface, to accommodate the double-bundle reconstruction (Figure 12).

**Figure 13:**
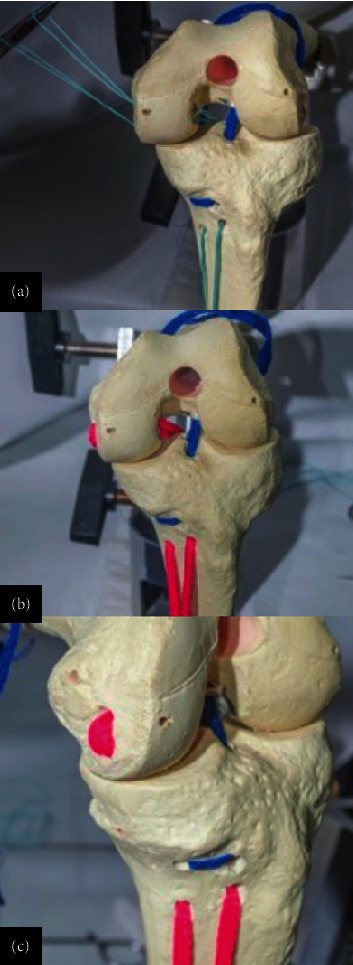
(a–c) Wires are retrieved through the anterior portals for the passage of graft sutures and traction through tibial tunnels.

The allograft was introduced into one femoral tunnel and passed subcutaneously through the second tunnel, creating a U-shaped configuration without the need for additional fixation (Figure 13).

**Figure 14:**
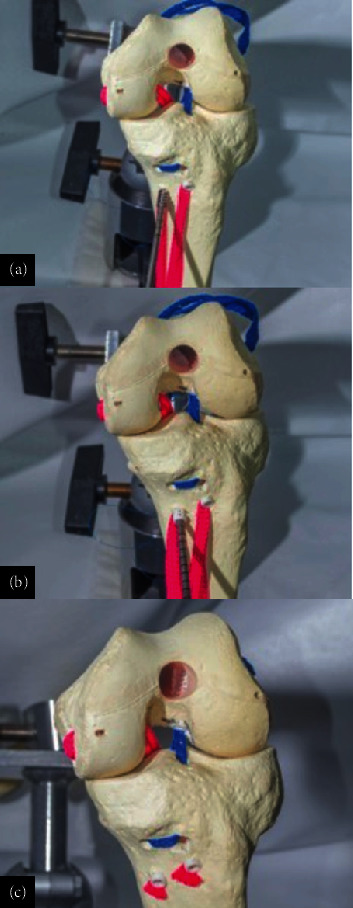
Graft with 25–30 cm length and 8–10 mm width per bundle. (a, b) The graft is introduced in one of the tibial tunnels and is passed subcutaneously through the tunnels in the U-shaped perforation, leaving the union in the femoral area. (c) Screw placement in tibial tunnels with knee fixation in extension.

The graft was guided through the posteromedial portal using cerclage wires in the tibial tunnels, where it is tensioned to minimize injury during angulation. Interference screws are placed at the extension to secure the graft (Figure 14).

### 3.3. Anatomic MPFL and MCL Reconstruction Technique

**Figure 15:**
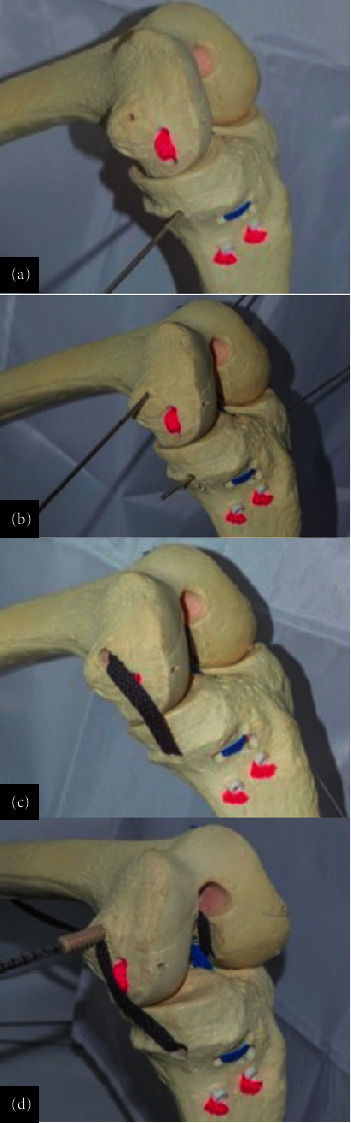
(a, b) Tibial tunnel drilling, (c) graft passage, and (d) fixation using bioabsorbable screws.

The reconstruction of the MPFL and MCL began with a small medial incision to expose the native attachment sites of both ligaments. The MCL reconstruction started with the identification of the tibial attachment site. A tunnel was drilled into the tibia to secure one end of the graft. The graft was then passed beneath the superficial MCL and anchored in the tibial tunnel with an interference screw. A second tunnel was drilled into the femoral attachment site, where the other end of the graft was fixed to restore medial knee stability. Careful attention was given to the tensioning of the graft to ensure adequate stability without causing knee stiffness or laxity (Figure 15).

**Figure 16:**
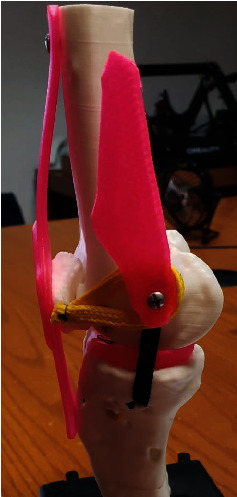
3D–printed anatomical model representation of surgical repair of MCL and MPFL.

For the MPFL a two-tailed technique using interference screw fixation reconstruction was performed, the patellar attachment was reinforced by passing the graft through two small tunnels drilled into the medial border of the patella, securing it with bioabsorbable screws. Proper tensioning was crucial to avoid overtightening, which could lead to patellar maltracking or recurrent dislocation. The graft was then tunneled and fixed at the femoral attachment point near the adductor tubercle, with the knee positioned in slight flexion during fixation to replicate the natural alignment and tension of the native MPFL (Figure 16).

### 3.4. Single-Tunnel Technique for PLC Reconstruction

**Figure 17:**
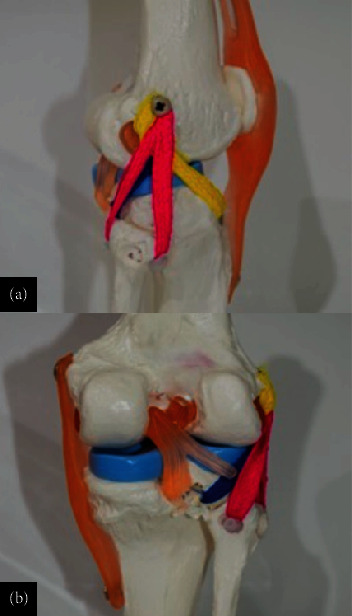
(a) Lateral and (b) posteroanterior images of PLC repair in the anatomical model.

The PLC reconstruction was initiated with a curvilinear skin incision on the lateral side of the knee, extending from the lateral femoral epicondyle to the fibular head. This incision exposed the biceps tendon, peroneal nerve, and other PLC structures. A tibialis posterior tendon allograft, prepared with Krackow sutures, was passed through a tunnel drilled into the proximal fibular head. The graft was then routed beneath the biceps femoris tendon and the iliotibial band toward the lateral femoral epicondyle. The limbs of the graft were crossed and fixed to the lateral femoral condyle using a biodegradable screw. The knee was held in valgus, internal rotation, and 20° of flexion during fixation to ensure that the graft remained isometric throughout its range of motion, which is essential for effective stabilization (Figure 17).

## 4. Results

**Figure 18:**
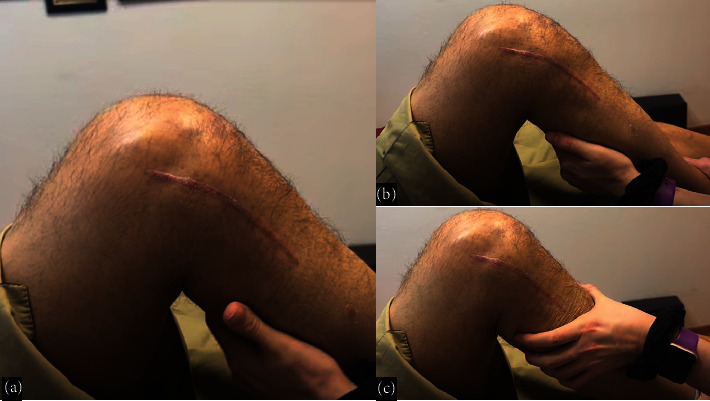
(a) Two months postoperative progress: The patient is walking independently without the need for support and is free of pain. (b) Physical examination demonstrates a negative anterior drawer test. However, (c) possible posterior sag may indicate some degree of residual posterior instability.

The patient exhibited a highly favorable outcome in early postoperative evaluation, with minimal edema and moderate pain, which responded well to analgesics. During the 30-day follow-up, adequate wound healing and full range of motion (0–135 degrees) of the knee were observed during physical examination, and stability of the joint was confirmed with the PSM app and knee stability maneuvers. The patient regained the ability to perform daily activities without pain or limitation (Figure 18).

## 5. Discussion

Historically, it has been shown that rotational stability is significantly better in patients with the anatomic double-bundle ACL reconstruction compared to the patients with the single-bundle procedure. As shown in a prospective study done around 2005–2015, the single-bundle ACL reconstruction resulted in significantly more graft failures during the 10-year follow-up than the double-bundle ACL surgery. At the 10-year follow-up, only 1 patient out of 30 patients had graft failure with the double-bundle technique leading up to revision ACL surgery, while with the single-bundle procedures, 10 patients out of 60 patients had revision ACL surgery because of the graft failure, showing its value as a tool in maintaining stability in MLKIs and return to activity [16, 17].

In the case of PCL double-bundle reconstruction, it significantly improved subjective outcomes and functional scores from preoperative states. Furthermore, the procedure substantially restored posterior tibial translation, as evaluated by kneeling stress radiographs, with a notable improvement in the side-to-side difference in posterior tibial translation postoperatively. These findings are consistent with biomechanical studies demonstrating that a double-bundle PCL reconstruction restores knee kinematics to near normal. However, it is important to note that mild residual posterior sag (1–2+) can persist in some cases, as observed in Figure 18(c). This common postoperative finding, while not indicative of graft failure, should be considered when assessing overall outcomes, as it does not typically hinder the functional and subjective improvements achieved by the procedure.

The use of a biomechanically validated technique with an 11-mm Achilles allograft and a 7-mm tibialis anterior allograft also provides a strong construct that improves objective stability. The literature describes other techniques that utilize smaller grafts with improved outcomes but are still leaning toward an improvement in general subjective and functional scores for the double-bundle PCL reconstruction compared to a single-bundle reconstruction [18].

In this case report, a multiligamentous knee injury was successfully addressed by combining PLC, MPFL, and MCL reconstruction with allograft and suture tenodesis performing U2 double-bundle reconstruction with allograft for the ACL and the PCL. The combination of these surgical techniques proved to be effective in the management of this complex injury.

Follow-up intervals included assessments at 2 weeks, 1 month, 3 months, 6 months, and 1 year, postoperatively. At each interval, the patient exhibited progressively improved function and stability, with no signs of graft failure or significant complications. By the 2-week follow-up, the patient showed minimal edema and moderate pain managed by analgesics. By 1 month, the patient had regained a full range of motion and reported a marked reduction in pain. The 3-month follow-up showed that the patient had returned to light activities without instability. By 6 months, the patient had resumed most daily activities with no pain, and at 1 year, the patient had returned to near-normal function, demonstrating the long-term efficacy of the combined surgical approach.

While the U-technique used in this case for double-bundle ACL and PCL reconstruction offers biomechanical advantages in restoring knee stability and function, it is important to acknowledge its potential limitations. One possible concern is the risk of attritional graft rupture as the graft “windshields” against the tibial cortex during knee motion. This repetitive friction could lead to gradual graft weakening or rupture over time, especially in active patients. Although this theoretical risk has been raised, there is limited data specifically addressing the long-term outcomes of the U-technique in relation to graft attrition. Future studies are needed to evaluate the durability of the grafts and whether modifications in the technique or additional protective measures are warranted to mitigate this potential issue.

It is important to highlight that early and exhaustive evaluations, along with timely surgical intervention and the meticulous techniques used in this case, were essential to achieve optimal results in the treatment of multiligamentous knee injuries. In summary, the combination of the fibular sling, MPFL and MCL reconstruction, and double-bundle U2 anatomical reconstruction for the ACL and PCL proved to be an effective strategy to address multiligamentous knee injuries, providing stability and functionality to the knee joint and improving quality of life.

## 6. Conclusion

This case demonstrates the successful management of a complex multiligamentous knee injury using a combination of the fibular sling, MPFL and MCL, and U2 double-bundle reconstruction of both the ACL and PCL. The early and thorough evaluation, followed by prompt surgical intervention, led to favorable outcomes, including minimal edema, manageable pain, and rapid restoration of knee function by the 30-day follow-up. This approach underscores the effectiveness of combining reconstruction techniques for restoring stability and functionality in complex MLKIs.

## Figures and Tables

**Table 1:** Schenck classification of knee dislocation.

KD I	Dislocation with a tear of only one cruciate ligament (ACL or PCL)
KD II	Tears to both cruciate ligaments (ACL and PCL), with functionally intact collateral ligaments.
KD IIIM	Tears of both cruciate ligaments and one collateral ligament (medial collateral ligament)
KD IIIL	Tears of both cruciate ligaments and one collateral ligament (lateral collateral ligament)
KD IV	Tears of both cruciate ligaments and both collateral ligaments
KD V	Fracture dislocation

## Data Availability

The data that support the findings of this study are available from the corresponding author upon reasonable request.
